# Cause exceptionnelle d'hémoperitoine spontané chez l'adulte: hémolymphangiome kystique rompu

**DOI:** 10.11604/pamj.2017.26.8.6533

**Published:** 2017-01-04

**Authors:** Boubacar Zan Traoré, Kamal Serrar, Omar Saoud, Khedid Yahia Zain El Abidine, Rachid Mohammed Chkoff, Rachida M'bida

**Affiliations:** 1 Clinique Chirurgicale des Urgences Viscérales, Hôpital Avicenne, CHU Rabat-Salé, Rabat, Maroc

**Keywords:** Hémolymphangiome kystique, hémopéritoine, adulte, rate, Cystic hemolymphangioma, hemoperitoneum, adult, spleen

## Abstract

L'hémolymphangiome est une tumeur bénigne résultant du développement anormal des vaisseaux lymphatiques associés à des malformations vasculaires. 50% à 60% de ces tumeurs sont présentes à la naissance. Les formes cervicales sont les plus fréquentes. Les localisations Abdominales sont très rares. Nous rapportons deux cas d'hémolymphangiome kystique de la rate révélé par l'hémoperitoine spontané admis en urgence au service des Urgences Chirurgicales Viscérales, avec une revue de la littérature. Il s'agit d'une patiente âgée de 50 ans et l'autre un jeune homme de 20 ans admis aux urgences pour respectivement, une sensibilité abdominale diffuse avec matité déclive des flancs et d'une distension abdominale dans un tableau de choc hémorragique, pâleur, pouls imprenable, stabilisé après une courte réanimation. La résection tumorale était complète chez tous les malades. Le diagnostic d'hémolymphangiome kystique de la rate était révélé à l'examen anatomopathologique des pièces opératoires. L'hémolymphangiome est une tumeur rare à pronostic favorable. Quelques cas de régression spontanée ont été décrits, mais l'évolution se fait classiquement vers une croissance lente de la tumeur. Il n'y a aucun pouvoir de dégénérescence maligne. Le traitement est chirurgical. Le pronostic est lié aux complications, à la qualité de l'exérèse chirurgicale et les récidives qui sont fréquents notamment en cas d'exérèse incomplète.

## Abstract (en)

Hemolymphangioma is a benign tumor resulting from the abnormal development of the lymphatic vessels associated with vascular malformations. 50% to 60% of these tumours are present at birth. Cervical forms are most frequent. Abdominales locations are very rare. We report, through a literature review, two cases of patients with cystic hemolymphangioma of the spleen revealed by spontaneous haemoperitoneum admitted to the Emergency Department of Visceral Surgery. The first case is a 50-year old patient and the second is 20-year old man admitted to the emergency department with diffuse abdominal pain associated with dullness to percussion on the dependent side and abdominal distension associated with haemorrhagic shock, pallor, unobtainable pulse, stabilized after a short resuscitation time respectively. All patients underwent complete tumor resection. The diagnosis of cystic hemolymphangioma of the spleen was based on anatomopathological examination of the surgical specimens. Hemolymphangioma is a rare tumor with a favorable prognosis. Some cases of spontaneous regression have been reported, but evolution is usually characterized by slow tumor growth. Malignant transformation never occurs. Treatment is surgical. Prognosis is subjected to complications, quality of the surgical excision and recurrences which are frequent especially after incomplete excision.

## Introduction

Le lymphangiome kystique est une tumeur bénigne développée aux dépends du système lymphatique comportant de multiples formations kystiques non communicantes [1]. Lorsque coexistent des malformations vasculaires, le terme d'hémolymphangiome est retenu [2, 3]. L'hémolymphangiome kystique est une tumeur considérée comme bénigne, rare, angio-dysplasique. Cinquante à 60% de ces tumeurs sont présentes à la naissance et 90% d'entre elles sont diagnostiquées durant les deux premières années de vie, évoluant par poussées hémorragiques d'autant plus fréquentes que le sujet est jeune et engageant potentiellement le pronostic vital.

## Patient et observation

Nous rapportons deux cas cliniques montrant la difficulté du choix thérapeutique en urgence et de son incidence sur la prise en charge chirurgicale.

### Observation N°1

**Figure 1:**
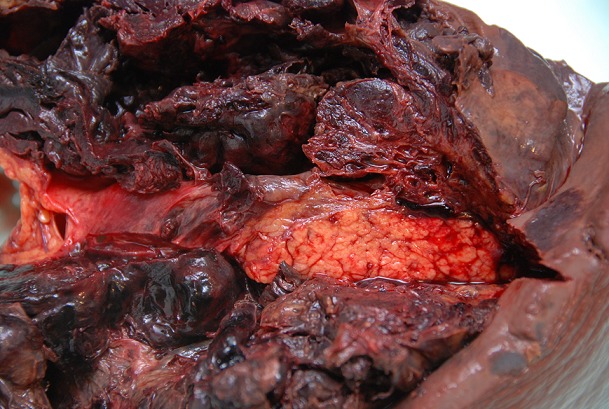
Aspect du hile splénique et de la queue du pancréas après la splénopancréatectomie caudale et évacuation des caillots et d’hématome

Une femme âgée de 50 ans, sans antécédents particulier, a été hospitalisée aux urgences pour un syndrome douloureux abdominal aigu de début brutal, associant douleurs épigastriques et de l'hypochondre gauche irradiantes dans le dos et des lombalgies avec anémie sévère. L'examen clinique révélait un abdomen globalement sensible et une matité déclive des flancs était notée. L'échographie abdominale montrait des images en faveur d'une tumeur du hile splénique avec épanchement péritonéal de moyenne abondance. Sur les résultats des prélèvements biologiques, il existait une anémie à 6,6 g/dl, une neutropénie à 9,09 10^3^l'hématocrite à 20,1% sans thrombopénie associée; l'alpha fœto-protéine, l'antigène CA 19-9 et les ACE étaient normaux. La TDM abdominale mettait en évidence une masse de l'espace intra péritonéal entre le hile splénique et la grosse tubérosité gastrique avec épanchement intra péritonéal en faveur d'une tumeur d'espace ayant fait évoqué une tumeur stromale. La laparotomie exploratrice était décidée pour hémoperitoine sur rupture tumorale. L'exploration abdominale après aspiration d'un litre d'hémoperitoine mettait en évidence une énorme masse encapsulée, contenant des caillots et d'hématome aux dépens du pédicule splénique. Cette masse adhère à la grande courbure gastrique et à la queue du pancréas. Une résection en monobloc de la masse emportant la rate et la queue du pancréas avait été réalisée (Figure 1). Le diagnostic d'hémolymphangiome kystique avait été confirmé par l'étude histologique de la pièce. L'évolution était favorable. Douze mois après, aucune récidive n'avait observée.

### Observation N°2

**Figure 2:**
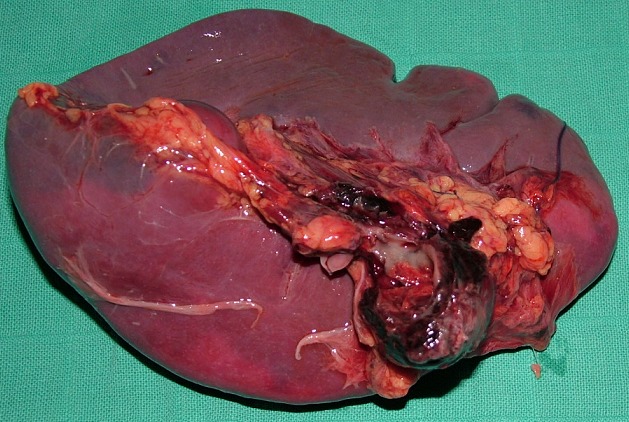
Hémolymphangiome kystique de petite taille du hile splénique

Malade de seconde mains, jeune homme de 20 ans, était opéré 24 heures avant son admission dans un tableau abdomen aigu avec état de choc sur hémoperitoine d'origine indéterminée dans une structure périphérique donc le compte rendu opératoire fait état d'une aspiration d'environ trois litres d'hémoperitoine à l'ouverture de la cavité péritonéale. L'exploration n'objectivait aucune lésion dans la cavité abdominale sauf un saignement en nappe entre la rate et la queue du pancréas. L'équipe de chirurgie avait réalisé un lavage de la cavité abdominale puis drainage. Les suites opératoires avaient été marqués par la persistance de l'hémorragie intra abdominale à travers les lames de drainage ce qui motiva l'évacuation du patient vers notre structure pour prise en charge. A son arrivé dans le service d'accueil des urgences, il était intubé et ventilé, en état de choc hémorragique, pâle, avec un pouls imprenable, un abdomen distendu. Après remplissage, le patient était rapidement transféré au bloc opératoire pour laparotomie exploratrice, l'exploration abdominale après aspiration de deux litres d'hémoperitoine mettait en évidence la présence de multiples caillots dans l'arrière cavité des épiploons, un saignement en nappe provenant d'une masse du hile splénique adhérente à la queue du pancréas et recouverte par des caillots. Nous avons réalisé une résection de la masse avec une splénectomie totale d'hémostase (Figure 2). Le diagnostic d'hémolymphangiome kystique avait été confirmé par l'étude histologique de la pièce. L'évolution etait favorable. Huit mois après l'exérèse chirurgicale, aucune récidive n'avait observée.

## Discussion

L'hémolymphangiome kystique est une tumeur dont l'origine est variable selon l'âge [1–3]. Elle est caractérisée par la présence de cavités vasculaires anastomotiques dont la lumière est vide et dont les parois sont fines, revêtues d'un endothélium mince avec des faisceaux musculaires en périphérie et îlots lymphoïdes. Les kystes peuvent être de taille variable et le contingent hémangiomateux plus ou moins important. Ce tissu tumoral englobe très souvent des structures nerveuses, comme cela a été le cas pour notre jeune patient. Chez l'adulte, il semble que l'hémolymphangiome kystique soit secondaire à un traumatisme ou à un obstacle survenu sur le système lymphatique quel qu'en soit l'étiologie. Il est alors parfois difficile de faire la différence entre lymphangiome, hématome, ectasie lymphatique ou néoplasie. Quelle que soit la technique chirurgicale choisie, une surveillance prolongée (échographie et tomodensitométrie abdominale) doit être systématique à la recherche d'une récidive [4].

## Conclusion

L'hémolymphangiome est une tumeur rare à pronostic favorable. Quelques cas de régression spontanée ont été décrits, mais l'évolution se fait classiquement vers une croissance lente de la tumeur. Il n'y a aucun pouvoir de dégénérescence maligne. Le traitement est chirurgical. Le pronostic est d'abord lié aux complications, à la qualité de l'exérèse chirurgicale qui conditionne les complications et les récidives. Les récidives sont fréquentes notamment en cas d'exérèse incomplète.
